# 

**Published:** 1863-07

**Authors:** 


New Series, Vol. 6, No. 7. Whole Series, Vol. 20, No. 7.

Box 1305

### THE CHICAGO

### MEDICAL JOURNAL.

DANIEL BRAINARD, M. D.,

J. ADAMS ALLEN, M. D.,

Editors and Proprietors.

JULY, 1863.

CHICAGO:

J. C. W. BAILEY, BOOK AND JOB PRINTER, 130 CLARK STREET.

1863.

